# Review: Clinical application of diffusion tensor imaging

**DOI:** 10.4103/0971-3026.38505

**Published:** 2008-02

**Authors:** Richa Trivedi, Ram KS Rathore, Rakesh K Gupta

**Affiliations:** Department of Radiodiagnosis, Sanjay Gandhi Postgraduate Institute of Medical Sciences, Lucknow, India; 1 Department of Mathematics and Statistics, Indian Institute of Technology, Kanpur, India

Diffusion tensor imaging (DTI), a relatively new MRI technique, has generated a tremendous amount of interest in the clinical and laboratory domains. This modality measures the Brownian motion of water molecules in tissue.[1] Two aspects of DTI, i.e., the microscopic length scale and orientation information render the modality very powerful. The microscopic length scale of water diffusion in tissue gives DTI microscopic spatial sensitivity, whereas the orientation information can be used to differentiate apparently homogenous white matter on conventional MRI into its constituent fiber tracts. Together, these two advantages have helped propagate the application of DTI in various pathologies.

To illustrate the ability of DTI to differentiate lesions from normal brain, this presentation reviews selected clinical applications of DTI.

## Physical Principles of DTI

Diffusion is a physical process that involves the translational movement of molecules via thermally driven random motion, the so-called Brownian motion. The underlying cellular microstructure of tissue complicates the situation and influences the overall mobility of the diffusing molecules by providing numerous barriers and by creating various individual compartments (e.g., intracellular, extracellular, neurons, glial cells, and axons) within tissue. In diffusion-weighted imaging (DWI), diffusion is described by using a scalar parameter, the diffusion coefficient *D*. However, in the presence of anisotropy, diffusion is characterized by a tensor, D, that describes local water diffusion. A tensor is a mathematical construct that describes the properties of an ellipsoid in 3-D space.

Quantities related to diffusion can be calculated from the tensor (matrix). Fractional anisotropy (FA) and mean diffusivity (MD) are the two most commonly used metrics for characterizing the tissue microstructural organization. FA (Equation 1) measures the degree of directionality of diffusion, while MD (Equation 2) describes the average diffusivity of water.

$$\mathrm{FA}\left(\lambda_{1},\lambda_{2},\lambda_{3}\right)=\frac{1}{\sqrt{2}}\sqrt{\frac{{\left(\lambda_{1}-\lambda_{2}\right)}^{2}+{\left(\lambda_{2}-\lambda_{3}\right)}^{2}+{\left(\lambda_{1}-\lambda_{3}\right)}^{2}}{\lambda_{1}^{2}+\lambda_{2}^{2}+\lambda_{3}^{2}}}$$

$$\mathrm{MD}=\frac{\lambda_{1}+\lambda_{2}+\lambda_{3}}{3}$$

## Potential Applications of DTI in the Central Nervous System

### Developmental disorders

DTI draws attention to developmental disorders, both congenital as well as natal, due to its potential for generating white matter tracts and aberrant connections in cases with disturbance of normal white matter development. In developmental central nervous system (CNS) disease, DTI demonstrates additional findings beyond those seen with conventional MRI and allows better understanding of a malformed brain.

#### Joubert syndrome:

**Figure 1 (A-F):**
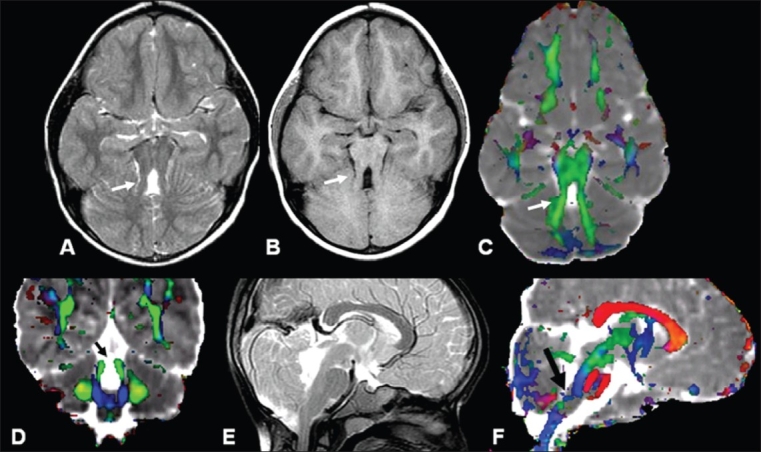
Joubert syndrome in a 2½-year-old girl with delayed development and hypotonia. Axial T2W (A) and T1W (B) MRI images show a ‘molar tooth’ appearance of the superior cerebellar peduncle (SCP) (arrow). Axial color-coded FA fused with MD map (C) shows uncrossed SCP fibers (arrow) that are clearer on the coronal image (arrow) (D). The sagittal T2W image (E) shows no obvious abnormality in the CST; however, the color-coded FA fused with MD map (F) shows thinning of the CST (arrow) at the level of the medulla

Joubert syndrome, a subtype of posterior fossa malformation, consists of vermian hypoplasia and derangement of the cerebellar-brainstem or cerebellar-cortical connections. The typical ‘molar tooth appearance’ of the superior cerebellar peduncle (SCP), with partial or complete absence of the vermis on MRI, is diagnostic of Joubert syndrome [Figure 1].[2]

#### Heterotopia:

In heterotopic gray matter, the arrested neurons due to a faulty migrational process might have some degree of directionality like normal white matter tracts and show increased anisotropy.[3]

#### Callosal agenesis:

**Figure 2 (A-F):**
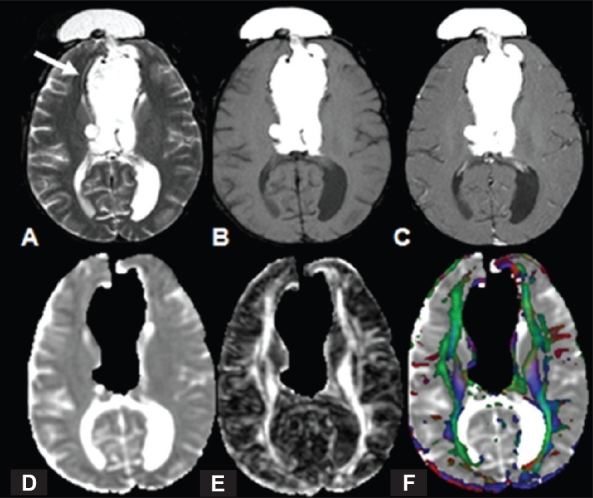
A 25-year-old woman with agenesis of the corpus callosum and an interhemispheric lipoma with subcutaneous extension. MRI shows an interhemispheric hyperintense mass protruding into the subcutaneous tissue (arrow) on T2W (A) and T1W (B) images, with no enhancement on the post-contrast T1W image (C). MD map (D) generated from the fat-suppressed DTI data, shows hypointensity in the lesion consistent with fat. FA (E) and color-coded FA fused with MD map (F) show agenesis of the corpus callosum

Agenesis of the corpus callosum (ACC) [Figure 2] is characterized by a ‘cartwheel configuration’ of the interhemispheric sulcal markings, absence of the cingulate gyrus, and colpocephalic features of the lateral ventricles.[4] DTI has shown a thick fiber bundle running anteroposteriorly (i.e., the Probst bundle) in these patients.

#### Cerebeller agenesis:[5]

**Figure 3 (A-L):**
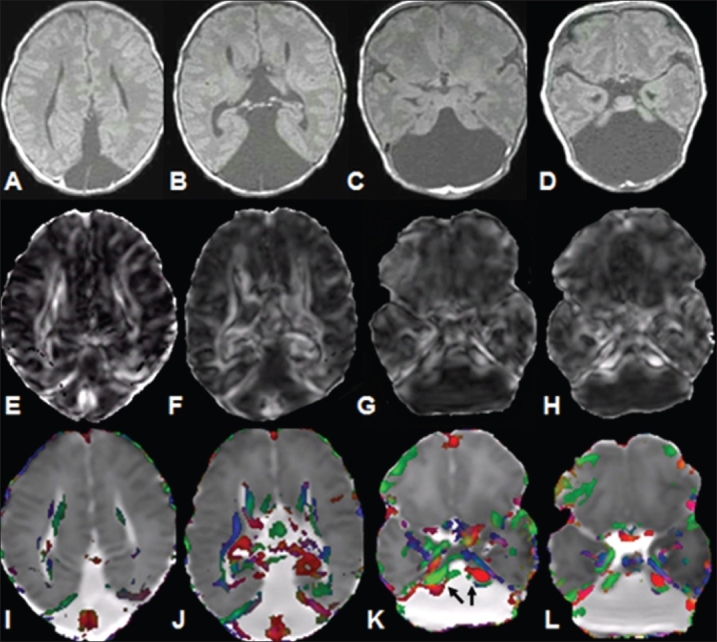
A 1½-month-old child with complete callosal and cerebellar agenesis. Consecutive T1W images (A-D) show absence of the cerebellum as well as the callosal fiber tracts (note the rudimentary pons in D). The posterior fossa is replaced by cerebrospinal fluid. FA maps (E-H) and color-coded FA fused with MD maps (I-L) show callosal as well as cerebellar agenesis, with large middle cerebellar peduncles showing high FA values (arrows)

Complete cerebellar agenesis [Figure 3] occurs during the early period of embryogenesis and is usually associated with severe motor dysfunction.

#### Polymicrogyria (PMG):

**Figure 4 (A-D):**
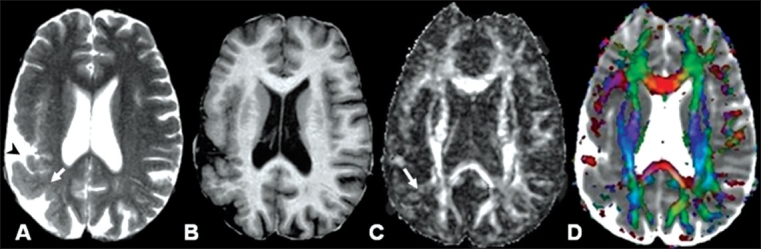
A 43-year-old man with a history of generalized seizures for the last 21 years. T2W axial image (A) through the lateral ventricles shows an abnormally thick cortex (arrow) with a bumpy appearance at the surface (arrowhead) and at the cortex-white matter junction in the right parietooccipital region. The abnormality is more prominently visible on the phase-sensitive T1 fluid-attenuating inversion recovery (FLAIR) axial image (B). The FA map (C) shows a diminished value (arrow) in the subcortical white matter subjacent to the polymicrogyric cortex, which is clearer on the color-coded FA modulated with MD map (D)

PMG, a surface area derivative anomaly, commonly manifests as a seizure disorder. MRI is routinely used to study these patients *in vivo*. Though conventional MRI provides detailed anatomic demonstration of these lesions, it does not demonstrate the true extent of the lesion. DTI has shown significantly decreased FA values [Figure 4] in the subcortical white matter subjacent to the polymicrogyric cortex, reflecting microstructural changes in the white matter, probably due to the presence of ectopic neurons.[6] An abnormality on DTI, seen beyond the margins of the obvious lesion on conventional MRI, may help in planning neurosurgical intervention.

#### Cerebral palsy (CP):

**Figure 5 (A-K):**
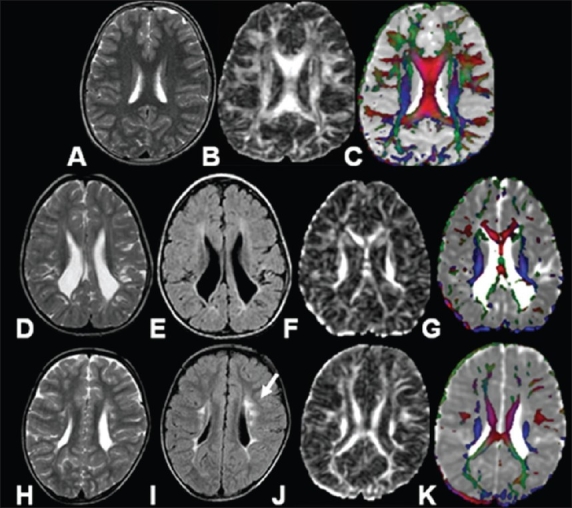
MRI images of an 8-year-old healthy control (A-C). T2W (A), FA map (B), and color-coded FA fused with MD map (C) show normal distribution of white matter. The MRI images of a 9-year-old child (D-G) with a clinical diagnosis of spastic hemiparesis, show nearnormal imaging on T2W (D) and FLAIR (E) sequences. The FA map (F) shows decreased FA values in the periventricular white matter region as compared to the control (C) that is clearer on the color-coded FA fused with MD map (G). MRI images of a 12-year-old child (H-K) with a clinical diagnosis of spastic hemiparesis. T2W (H) and FLAIR (I) images show hyperintense lesions (arrow) in the periventricular white matter. The FA map (J) shows decreased intensity in the periventricular white matter that is clearer on the color-coded FA fused with MD map (K)

CP is a nonprogressive disorder of diverse etiology, characterized by varying motor dysfunction. The most common cause of childhood CP is hypoxic brain injury and periventricular leukomalacia (PVL) in premature neonates. An impairment of the corticospinal tracts (CST) is believed to be responsible for the motor dysfunction. MRI is useful for investigating the cause and timing of injury in children with CP[7] and has shown pathology in almost 70-90% of CP patients. A few recent DTI studies[8,9] have demonstrated decreased FA values in the posterior limb of the internal capsule and CST [Figure 5], even in patients with near-normal conventional imaging. Decreased CST FA values in CP patients may help in better defining the clinical outcome in these patients.

### Ischemic brain injury

A reduction in the delivery of oxygen and nutrients to the brain parenchyma secondary to obstructed blood flow results in cerebral ischemia. The rapid failure of high-energy metabolism and associated ionic pumps during acute cerebral ischemia leads to intracellular migration of sodium and calcium. The subsequent influx of osmotically obligated water results in cytotoxic edema.

#### Hypoxic-ischemic encephalopathy (HIE):

**Figure 6 (A-S):**
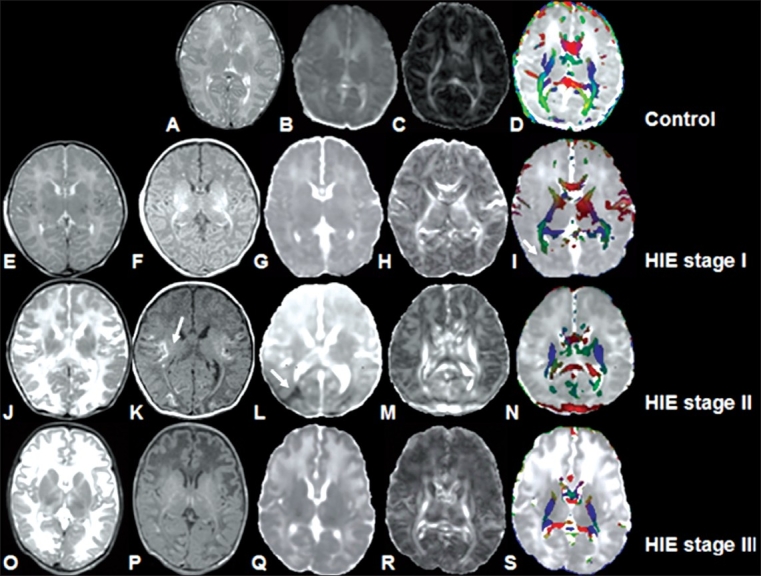
9-day-old normal term baby (A-D). T2W (A), MD (B), FA (C), and color-coded FA fused with MD map (D) show normal distribution of white matter. **Stage I HIE (E-I):** T2W (E), T1W (F), and MD (G) images from a 6-day-old term neonate appear normal. FA map (H) shows decreased FA values in the major white matter tracts compared to control (C) that are clearer on the color-coded FA fused with MD map (I). **Stage II HIE (J-N):** The T2W image (J) from a 4-day-old neonate appears normal. The T1W (K) image shows cortical and subcortical hyperintense lesions (arrow). The MD map (L) shows decreased MD values in the cortical and subcortical regions (arrow). The FA map (M) shows decreased FA values compared to both control (C) and stage I HIE (H) images, which are clearer on the color-coded FA fused with MD map (N). **Stage III HIE (O-S):** T2W (O), T1W (P), and MD map (Q) from a 10-day-old term neonate appear normal. However, the FA (R) and color-coded FA fused with MD map (S) show decreased FA in the major white matter tracts compared to control (C), HIE I (H), and HIE II (M) images

Permanent damage to neuronal cells caused by hypoxic-ischemic injury may result in neonatal death or be manifested later as CP or impaired cognition.[10] Due to incomplete myelination and the higher brain water content[11] in term neonates, conventional MRI is limited in its ability to detect the presence and extent of hypoxic-ischemic injury in stage I HIE; structural changes usually manifest after 4-8 months in infants with stage I and II HIE.[12] In term neonates with HIE, the detection of injury on DWI is dependent on the timing of imaging after injury. DTI imaging shows abnormal FA and mean diffusivity (MD) values in HIE patients even when they have near-normal conventional imaging [Figure 6].[13] An altered pattern of age-related changes in FA and MD values, which may help in earlier and more accurate assessment of microstructural damage in this setting,[13] has also been demonstrated using serial DTI scans.

#### Adult stroke:

**Figure 7 (A-E):**
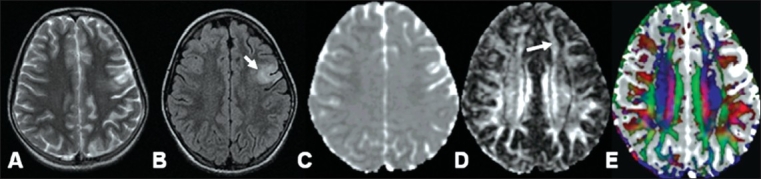
T2W (A), FLAIR (B), and MD map (C) images show an infarct in the left frontoparietal region (arrow) in a 30-year-old woman. The corresponding FA map (D) and the color-coded FA fused with MD map (E) images show low FA in the infarcted region (arrow) as well as in the area subjacent to the infarct, which appears normal on conventional imaging 6 weeks after the onset of stroke

Diffusion imaging is a valuable clinical tool in the assessment of stroke because of its high sensitivity during the hyperacute period[14] and its ability to distinguish acute infarcts from chronic infarcts. In the transition from acute to subacute to chronic stroke, the MD first decreases in the acute phase and then renormalizes and subsequently increases. On the other hand, diffusion anisotropy measures (e.g., FA) decline and remain low in chronic infarcts. Several experimental and human stroke DTI studies[15,16] in acute as well as chronic stroke settings have shown decreased FA values in infarcted areas as well as in the adjacent regions [Figure 7].

### Neuroinfection

Though the major application of DTI is to investigate white matter abnormalities in various neuropathologies, Gupta and his coworkers have demonstrated its use in many different infections.[17–21]

#### Brain abscess:

**Figure 8 (A-F):**
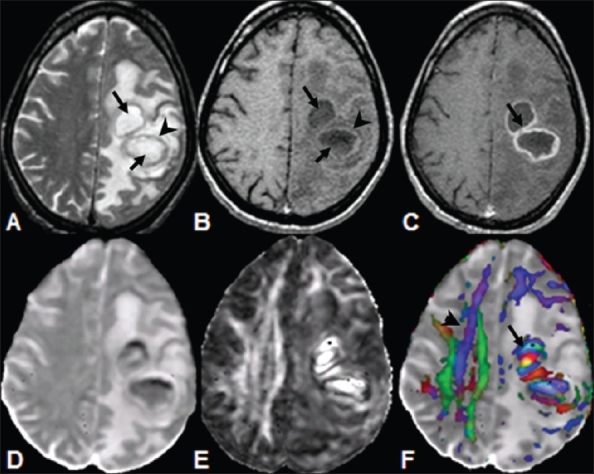
A 37-year-old man with multiple pyogenic brain abscesses in the left frontoparietal lobe. The axial T2W image (A) shows well-defined hyperintense lesions (arrows) with hypointense walls (arrowhead). The lesions appear hypointense (arrows) on the axial T1W image (B) with isointense walls (arrowhead). On the post-contrast T1W image (C) the lesions showring enhancement (arrow). The MD map (D) shows heterogeneous distribution of hypointensity. The FA (E) and color-coded FA fused with MD (F) maps shows orientation in the abscess cavities (arrow) similar to that observed in the contralateral corona radiata fiber tracts (arrowhead)

**Figure 9 (A-F):**
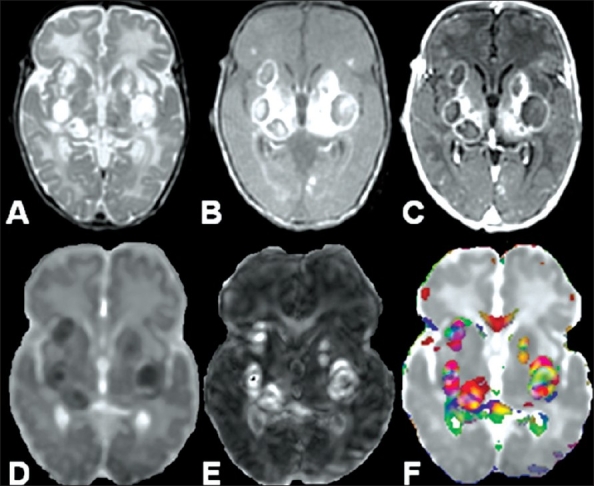
A 15-day-old neonate with multiple pyogenic brain abscesses. The axial T2W image (A) shows well-defined, hyperintense lesions with hypointense walls. The lesions appear hypointense on the axial T1W image (B). On the post-contrast T1W image (C) the lesions show ring enhancement. The abscess cavity appears hypointense on the MD map (D). The FA (E) and color-coded FA fused with MD (F) maps show orientation in the abscess cavities

Increased FA values, comparable to those seen in white matter, are seen in abscess cavities [Figures 8 and 9].[18] These are due to the presence of oriented neuroinflammatory molecules, as shown by experimental *ex vivo* imaging of cell lines treated with heat-killed *Staphylococcus aureus*.[21] FA may be used as a noninvasive surrogate marker for disease activity at the site of the local infective process.

#### Meningitis:

**Figure 10 (A-F):**
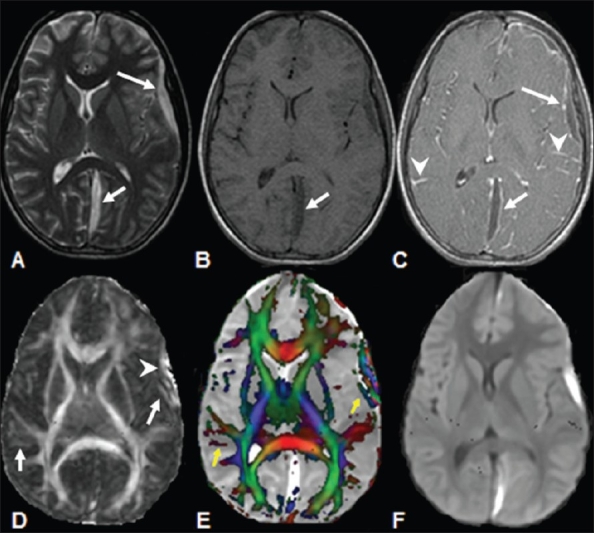
A 14-year-old patient with meningitis with an associated subdural collection of pus in the frontotemporal region and interhemispheric fissure. The T2W axial image (A) shows hyperintensity in the frontotemporal region and interhemispheric fissure (arrows), which appears hypointense (arrows) on the T1W image (B) and shows wall enhancement (arrows) on the post-contrast T1W (C) image. Meningeal enhancement (arrowheads), consistent with meningitis, is also seen on this image. The FA map (D) shows increased FA values (arrowhead) in the subdural collection as well as in the cortical grey matter region (arrows), which enhances on the post-contrast T1W image. The abnormality (yellow arrow) is more clearly visible on the color-coded FA map (E) modulated by the principal eigenvector. The DWI image (F) shows areas of restricted diffusivity in the subdural collection

Gadolinium (Gd)-DTPA MRI can detect abnormal meningeal enhancement.[22] High FA values in enhancing and nonenhancing cortical ribbons in adults [Figure 10] as well as in neonates with bacterial meningitis have been shown[19,20] as compared to age-matched controls. This may be due to oriented inflammatory cells in the subarachnoid space as a result of an up-regulated immune response in meningitis. Increased FA values in the enhancing as well as nonenhancing cortical regions suggest diffuse inflammatory activity in the pia-arachnoid in these patients. FA may be a better indicator of active and diffuse meningeal inflammation than post-contrast T1W imaging.

The periventricular white matter of the neonatal brain is known to be vulnerable to oxidative and hypoxic-ischemic injury secondary to neuroinfections. A recent DTI study has shown decreased FA values in the normal appearing periventricular white matter of neonates with bacterial meningitis compared to age/sex-matched healthy controls.[20]

#### Subacute sclerosing panencephalitis (SSPE):

**Figure 11 (A-I):**
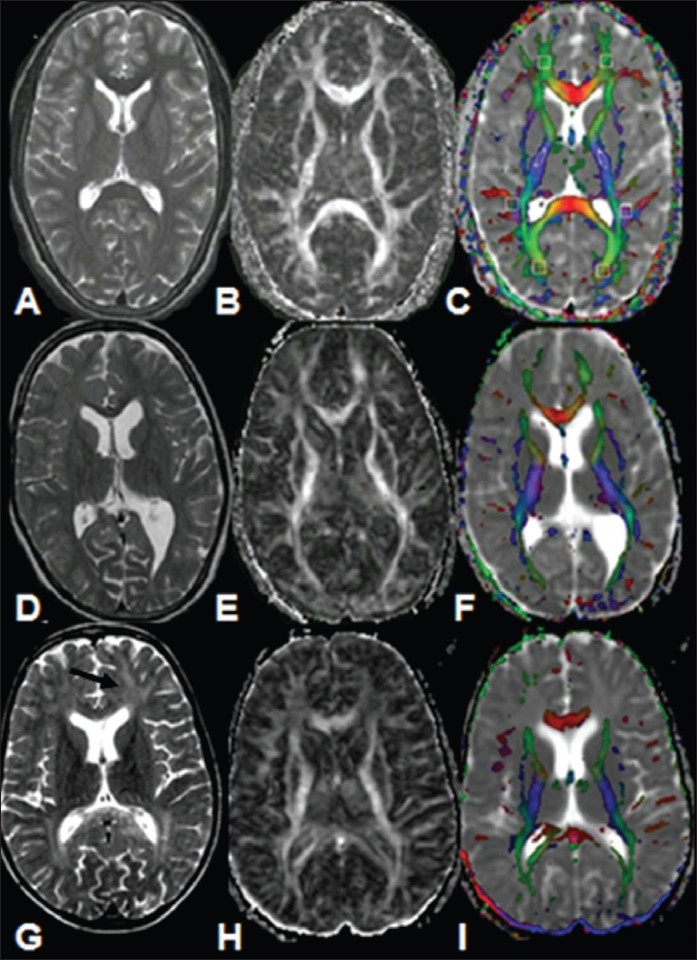
A 6-year-old healthy control (A-C). T2W image (A) and FA (B) and color-coded FA fused with MD (C) maps through the lateral ventricles shows normal distribution of white matter. A 12-year-old boy (D-F) with clinical findings of SSPE shows a normal appearance on the T2W image (D). The FA map (E) shows bilateral significantly low FA values in the white matter. The color-coded FA fused with MD map (F) shows the abnormality more clearly. A 7-year-old boy (G-I) with SSPE shows hyperintensities on the T2W (G) image (arrow) in both frontal and occipital regions. The FA map (H) shows widespread bilateral abnormal white matter and thinning of the genu and splenium of the corpus callosum. The color-coded FA fused with ADC map (C) shows the abnormality more clearly

SSPE is a rare progressive degenerative disease. It is caused by persistent infection by a defective measles virus. Imaging in these patients is not used for diagnosis but for following the course of disease. To date, no significant correlation between conventional MRI and clinical staging has been demonstrated; even severely affected patients may show a normal MRI study.[23] DTI has shown decreased FA and increased MD values in the parietooccipital white matter [Figure 11] even in those with near-normal conventional imaging.[17]

#### HIV infection:

During the early stages of infection, HIV-1 enters the central nervous system and preferentially affects the subcortical white matter. Immunohistochemical and *in situ* hybridization studies in patients with HIV encephalitis have shown HIV-1 infected macrophages and multinucleated giant cells preferentially invading the cerebral white matter, the corpus callosum, and the internal capsule.[24] White matter involvement is detectable in the form of vasculitis and gliosis even in asymptomatic HIV-1 positive patients.[25] DTI studies in HIV patients with near-normal conventional imaging have demonstrated reduced FA values in white matter tracts. Fillipi *et al*. have demonstrated a linear relation between the viral load and DTI measures in white matter.[26]

### Radiation oncology

Radiotherapy causes injury to normal brain that may not be detected on conventional imaging.[27] Quantitative evaluation of changes in normal-appearing white matter (NAWM) of brain tumor patients receiving radiotherapy has been described using DTI metrics. Also reported are a significant reduction in FA and increase in MD in the brains of children (aged between 3-14 years and treated for medulloblastoma) and adults (with diverse brain tumors) who received various combinations of chemotherapy and radiotherapy.[28,29] In a recent study, FA values also demonstrated a differential effect in the frontal lobe, as compared to the parietal lobe, in medulloblastoma survivors.[30] Decreased FA values have been demonstrated in the NAWM of adult patients with low-grade gliomas, for dose bins of >55 Gy, 50-55 Gy, and 45-50 Gy, 3 months post-radiotherapy, suggesting that the threshold dose limit for changes in NAWM using DTI is 55-50 Gy.[31]
